# Increased Pentraxin 3 Levels Correlate With IVIG Responsiveness and Coronary Artery Aneurysm Formation in Kawasaki Disease

**DOI:** 10.3389/fimmu.2021.624802

**Published:** 2021-04-12

**Authors:** Toshiyuki Kitoh, Tsuyoshi Ohara, Taichiro Muto, Akihisa Okumura, Reizo Baba, Yusuke Koizumi, Yuka Yamagishi, Hiroshige Mikamo, Kenji Daigo, Takao Hamakubo

**Affiliations:** ^1^ Laboratory of Pediatrics, School of Pharmacy, Aichi Gakuin University, Nagoya, Japan; ^2^ Department of Pediatrics, School of Medicine, Aichi Medical University, Nagakute, Japan; ^3^ Department of Lifelong Sports and Health Sciences, College of Life and Health Sciences, Chubu University, Kasugai, Japan; ^4^ Department of Clinical Infectious Diseases, School of Medicine, Aichi Medical University, Nagakute, Japan; ^5^ Department of Protein-protein Interaction Research, Institute for Advanced Medical Sciences, Nippon Medical School, Tokyo, Japan

**Keywords:** pentraxin 3, Kawasaki disease, coronary aneurysm, intravenous immunoglobulin therapy, N-terminal pro-brain natriuretic peptide, presepsin, systemic vasculitis, coronary artery abnormalities

## Abstract

Kawasaki disease (KD) is a febrile disease of childhood characterized by systemic vasculitis that can lead to coronary artery lesions (CAL). This was a prospective cohort study to determine the levels of the pentraxin 3 (PTX3), soluble CD24-Subtype (Presepsin) and N-terminal pro-brain natriuretic peptide (NT-pro BNP) in consecutive KD patients. From January 2013 to March 2015, all patients with KD admitted to Aichi Medical University Hospital who provided consent had their plasma saved before IVIG administration. In total, 97 cases were registered. 22 cases of incomplete KD were excluded from the outcome analysis. The total 75 cases were used for statistical analyses. A PTX3 threshold of >7.92 ng/ml provided a specificity of 88.5 %, a sensitivity of 94.4 %, and a likelihood ratio as high as 15.92 for the diagnosis of KD compared with febrile non-KD controls. Although an echocardiographic diagnosis of CAL in the early course of the disease was confirmed in 24 cases, it was not in the remaining 51 cases. Neither NT-proBNP nor Presepsin had statistical significance for the prediction of the echocardiographic CAL diagnosis. Only PTX3 was significantly predictive of the echocardiographic CAL diagnosis (p=0.01). The PTX3 level was significantly higher in the intravenous immunoglobulin (IVIG) non-responders (45.9±7.45) than in the IVIG responders (17.0 ± 1.46 ng/ml) (p< 0.001). The PTX3 level also correlated with the number of IVIG treatment courses needed to resolve fever (R² =0.64). Persistent CAL (pCAL) formation was observed in three cases; one of aneurysm only and two aneurysms with dilatations. The patients with pCAL had significantly higher PTX3 levels (85 ± 8.4 ng/ml) than patients without pCAL (22 ± 2.2 ng/ml) (p< 0.0001). In terms of pCAL prediction, the area under the curve (AUC) of receiver operating characteristic ROC curve of PTX3 was 0.99, and it was significantly greater than that of Presepsin (0.67) or NT-proBNP (0.75). PTX3 is a soluble pattern recognition molecule that acts as a main component of the innate immune system. These data suggest that PTX3 can be utilized as a definitive biomarker for the prediction of IVIG resistance and subsequent CAL formation in patients with KD.

## Introduction

Kawasaki disease (KD) is an acute inflammatory disorder of unknown origin associated with medium-sized vessel vasculitis (1). However, inflammation of medium-sized arteries throughout the body, particularly of the coronary arteries, can occur during the acute phase of KD (2), and coronary artery aneurysms develop in the subacute phase (3). Coronary artery lesions (CAL), including dilations and aneurysms, respectively manifesting in the acute and transient form or persistent chronic form (designated as persistent CAL; pCAL), constitute the most serious complication. PCALs are the most common cause of acquired heart disease in children in developed countries (4, 5). Coronary artery aneurysms develop in 15–25% of untreated children and may lead to myocardial infarction, sudden death, or ischemic heart disease (6). Without treatment, mortality may approach 1%, usually within six weeks of onset (7).

Timely initiation of treatment with intravenous immunoglobulin (IVIG) has reduced the incidence of coronary artery aneurysms from 25% to 4% (8, 9). Even with treatment in the acute phase of KD with IVIG and aspirin, up to 5% of patients will develop serious and potentially life-threatening cardiac complications (10). Several scoring systems have been developed to identify children at highest risk for coronary artery abnormalities (11–13) and has been confirmed as a powerful predictor of coronary artery aneurysms in various Japanese studies. In other countries, however, it is not followed due to the imperfect performance of scoring systems (14).

Although the most appropriate treatment for patients who fail to respond to IVIG remains unclear, several suggestive trials have been reported. Severe KD that is resistant to IVIG may benefit from intravenous pulse corticosteroid therapy (15), infliximab infusion, or plasma exchange (PE) (16, 17). If a reliable biomarker were to be identified that would make it possible to distinguish the patients with KD at high risk for CAL formation, prompt initiation of second line therapy would lessen the risk of subsequent pCAL formation.

We hypothesized that the plasma concentrations of pentraxin 3 (PTX3), N-terminal pro-brain natriuretic peptide (NT-proBNP) or the soluble CD14 subtype (Presepsin) in patients with KD might be increased and thus reflect the severity of KD. We have expected that at least one of these might serve as a useful biological marker of CAL formation and prove useful as an additional part of the scoring systems. PTX3 is one of the pathogen recognition receptors (18). NT-proBNP is conventionally utilized as a biomarker to predict CAL formation. Presepsin represents a newly emerging sepsis marker. We examined the correlation between the measured values of these candidate markers and the rate of IVIG resistance as well as the incidence of coronary artery lesions.

## Materials and Methods

### Study Population and Laboratory Evaluation

The study was carried out in accordance with the principles of the Declaration of Helsinki and approved by the Institutional Review Board of the Aichi Medical University Hospital. Written informed consent was obtained from the subjects or their parents. All children recruited in this study were admitted to Aichi Medical University (AMU) in the period between January 2013 and March 2015. The children were evaluated by experienced clinicians at AMU and fulfilled the diagnostic criteria for KD as endorsed by the Diagnostic Guidelines for Kawasaki Disease (5th revision) (19). All of the KD patients were treated with oral aspirin (30 mg/kg per day) and IVIG (2 g/kg per day). 115 cases were registered in total. 18 cases that presented with fever, but with symptoms that were not compatible with KD, were excluded. 22 cases of incomplete KD were also excluded from the outcome analysis. 41 boys and 34 girls fulfilling the standard diagnostic criteria for KD were analyzed in the study. The plasma samples for research were collected from a total of 75 patients in three phases: 75 patients at the time of KD diagnosis/disease onset and prior to IVIG treatment (acute phase), 60 patients at 1 to 2 weeks following IVIG treatment (sub-acute phase), and 54 patients at 3 weeks to 4 months following disease onset (convalescent phase). There were 61 patients whose samples were obtained in all three phases. Along with these parameters, additional clinical laboratory data [i.e., ESR, CRP, the white blood cell (WBC) count, absolute lymphocyte count (ALC), absolute neutrophil count (ANC), platelet (PLT) count, D-dimer and alanine aminotransferase (ALT)] were evaluated for each phase of the disease. Samples from 18 children with fever, but with symptoms incompatible with KD, were used as febrile non-KD controls. Control plasma was also obtained from 20 non-febrile healthy control subjects during the course of the pre-operative evaluation.

### Echocardiogram Measurements and Failure to Respond to IVIG

A complete echocardiogram was conducted as part of the standard diagnostic evaluation of KD for all patients in each phase of the disease according to the “Guidelines for Diagnosis and Management of Cardiovascular Sequelae in Kawasaki Disease” (JCS 2013) (20).

### Quantification of Biomarker Candidates

Venous blood samples were collected in sterile tubes containing EDTA-potassium and centrifuged at 2000 g for 10 minutes at 4°C. The plasma samples were extracted from the aliquots and were immediately frozen and stored at −80°C until analysis. The plasma PTX3 and N-terminal pro-brain natriuretic peptide (NT-proBNP) levels were determined by enzyme-linked immunosorbent assay (ELISA) (Perseus Proteomics Inc. Tokyo, Japan) (21) and Biomedica Medizin produkte GmbH & Co KG, Wien, Germany), respectively following the manufacturers’ instructions. Plates were analyzed in duplicate on a Spark™ 10M multimode microplate reader (Tecan Trading AG, Switzerland). The plasma Presepsin level was determined using a chemiluminescent enzyme immunoassay (CLEIA) (Pathfast™, Chemical Medience Corporation, Tokyo, Japan) (22).

## Statistical Analysis

Statistical analysis was performed using the statistical software package Prism 8.0 for Windows (GraphPad, San Diego, CA, USA). Student's t-test was used for comparison between normally distributed continuous data. One-way or two-way ANOVA followed by Bonferroni’s comparison was used as a post hoc test to evaluate the statistical difference between more than two groups. The t-test analysis was used to evaluate the statistical difference between two groups. Non-parametric analysis using the Mann–Whitney U test was utilized for continuous data that did not follow a normal distribution. The relationship between quantitative variables was assessed using the Spearman correlation coefficient. Statistical significance was taken as p < 0.05. To compare the power of plasma biomarker candidates to predict IVIG unresponsiveness or CAL formation, receiver-operating curves (ROCs) were plotted and area under the curves (AUCs) were calculated. The optimal cut-off points were determined using ROC curves to maximize the sensitivity and specificity.

## Results

### Clinicoepidemiological Characteristics of the KD Patients

The plasma samples used for research were collected from 75 children enrolled during the study period. One boy had two episodes of KD and one girl had three episodes of KD. Seven cases of KD received no IVIG; 45 cases, one dose; 19 cases, two doses; 4 cases, three doses (**Table 1**). Among the 68 IVIG-treated KD patients, 46 cases were IVIG responders and 22 cases were IVIG non-responders. Infliximab was administered to two patients after two doses of IVIG. Plasma exchange was performed in two patients after three doses of IVIG. An elective second line use of steroids was not employed in this cohort. Of the 75 patients, 24 had transient coronary artery lesions detected by echocardiography. Ultimately, three patients had persistent CAL sequelae according to echocardiography and 72 did not.

**Table 1:** Clinical characteristics of patients with Kawasaki disease.

Characteristic	No. of patients (%)
Total	75
Age (months)	
Median	27
Range	3–114
Gender (male/female)	39/36
Intravenous gamma globulin	
Yes	68 (90.7)
one dose of IVIG	45
two doses of IVIG	19
three doses of IVIG	4
No	7 (9.3)
IVIG treated	68
IVIG responder	46 (67.6)
IVIG non-responder	22 (36.4)
acute coronary artery abnormalities	24 (32.0)
chronic coronary artery abnormalities	3 (4.0)
Aneurysms	3 (4.0)
Aneurysms & Dilatations	2 (2.7)
Follow up (months)	
Median	18
Range	5–34
Outcome	
Complete recovery without sequelae	72 (96.0)
Persistent aneurysm	3 (4.0)

### Elevated Levels of PTX-3, NT-proBNP During Acute KD

**Figure 1:**
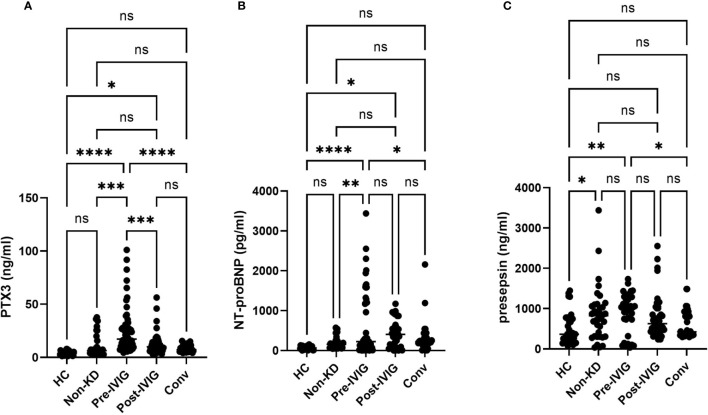
Plasma levels of PTX3, NT-proBNP and Presepsin during the disease course of KD. Unpaired Student t-test. Comparison of the level of PTX3 **(A)**, NT-proBNP **(B)**, and Presepsin **(C)** in healthy non-febrile controls, non-KD febrile controls, and KD. Between-group differences were determined using ANOVA and logical regression analysis. A: Circulating levels of PTX3 throughout KD. B: Circulating levels of NT-proBNP throughout KD. C: Circulating levels of Presepsin throughout KD. ns, not significant; *p < 0.05, **p < 0.01, ***p < 0.005, ****p < 0.001. HC, healthy controls; Non-KD, non KD febrile disease; at the time of onset of KD or before IVIG (Pre-IVIG), after IVIG (Post-IVIG); Conv, convalescent.

The KD patients were evaluated for the respective circulating levels of PTX3, NT-proBNP and Presepsin throughout the clinical course of the disease. We set three sampling time points during the disease course: at the time of onset of KD or before IVIG (Pre-IVIG), after IVIG (Post-IVIG) and during convalescence period after resolution of the fever (Conv). The PTX3 and NT-proBNP levels were significantly higher in the before-IVIG group compared with febrile non-KD controls (**Figures 1A, B**). Both were significantly decreased in the KD patients after IVIG treatment. The levels of Presepsin in the patients with KD were not significantly higher than in the febrile non-KD controls (**Figure 1C**). Plasma Presepsin was not upregulated during the acute phase.

The PTX3 levels of healthy controls and febrile non KD patients on admission were 3.8 ± 0.35 ng/ml (n=20) and 11.8 ± 2.2 ng/ml (n=18), respectively. The PTX3 levels in the KD patients (33.4 ± 7.3 ng/ml) were significantly higher than those in the non-KD febrile controls (11.8 ± 2.2 ng/ml; p < 0.001). The PTX3 threshold of >7.92 ng/ml provided a specificity of 88.5 %, a sensitivity of 94.4 %, and a likelihood ratio as high as 15.92 for the diagnosis of KD compared with the febrile non-KD controls. The high levels of PTX3 noted before IVIG treatment decreased after treatment (Pre-IVIG, 33.4 ± 7.3 ng/ml; Post-IVIG, 9.64 ± 3.61 ng/ml; **Figure 1A**).

### Plasma PTX3, NT-proBNP and Presepsin Levels and Coronary Artery Lesions in Acute Phase KD 

In the acute phase of KD, the high echo density of the perivascular area of the coronary artery or coronary artery dilatation (echocardiographic CAL diagnosis; echoCAL) was detected in 23 cases by echocardiography (**Table 2**). There were no significant differences in the age or the ratio of males to females between the echoCAL (–) and echoCAL (+) groups. No significant difference was found for the day of illness at the time of IVIG administration (5.4 [3-8] and 5.3 [3-12] day) and the sampling day of the illness (4.5[1-7] and 4.7 [2-12] day). There were no significant differences in any other of the examined variables, such as WBC count, serum sodium, CRP, D-dimer, ALT and Albumin levels between the 2 groups. The result of using an independent t-test indicated that the p-values of NT-proBNP and Presepsin were both 0.05 or higher, and there was thus no statistical significance. The p-value of PTX3 was considered significant at 0.01

**Table 2:** Demographic, laboratory characteristics of patients at the time of diagnosis in the groups without CAL and with CAL.

	CAL(-)	CAL(+)	P value
	(n = 51)	(n = 24)	
Age (m)	20.95±2.9	19.38±4.1	0.45
	[3-72]	[2-78]	
Male sex (%)*	0.4	0.4	0.92
Day of illness at the time of IVIG administration (d)	5.4±1.9	5.3±1.2	0.91
	[3-8]	[3-12]	
Sampling day of illness (d)	4.5±1.3	4.7±1.8	0.61
	[1-7]	[2-12]	
IVIG non-responder (%)*	12±0.24	10±0.42	0.04
WBC count (103/mL)	13329±4400	13225±3716	0.92
	[3090-24900]	[6000-23900]	
Sodium (mEq/L)	136.5±2.89	135.8±2.93	0.23
	[130-144]	[129-141]	
CRP (mg/dL)	5.13±3.31	6.22±4.24	0.23
	[0.36-12.88]	[0.99-15.88]	
D-Dimer (μg/mL)	2.07±2.26	2.20±1.09	0.85
	[0.9-9.03]	[1.01-5.21]	
ALT	62.2±9.6	135.8±18.5	0.27
	[7-422]	[7-807]	
ALB	3.82±0.33	3.76±0.30	0.42
	[2.9-4.5]	[3.1-4.5]	
NT-proBNP (pg/mL)	453.2±199.6	831±443.5	0.05
	[7.4-1659]	[6.2-3437]	
Presepsin (pg/mL)	482.7±237.9	818.2±455.6	0.04
	[74-1943]	[103-3437]	
PTX3 (ng/mL)	18.16±11.6	33.21±14.9	0.01
	[3.22-5383]	[4.4-101]	

Presented as the mean ±SD with the 5th and 95th percentiles in square brackets. Mann-Whitney U test. *2-sided χ2 test. Echo CAL, high echo density of the perivascular area of the coronary artery or coronary artery dilatation.

### The PTX3, NT-proBNP and Presepsin Levels Used to Predict IVIG Resistance/Non-Responders

IVIG resistance was defined as a persistent or recurrent fever for more than 48 h after completion of the initial IVIG treatment. Failure to respond is typically defined as persistent or recrudescent fever ≥24 hours after completion of the initial IVIG infusion. Plasma levels were determined in the IVIG responders (n = 46) and IVIG non-responders with KD (n = 22) in evaluating the IVIG response. Twenty-two of the 68 IVIG-treated patients were non-responders (IVIG res (-)) (**Table 3**). There was no significant difference in sex, the day of illness at the time of IVIG administration, nor the sampling day of illness between the IVIG res (+) and IVIG res (-). The number of patients who developed CAL was not significantly higher in the IVIG res (+) group (41% in IVIG res (+) vs 50% in IVIG res (-) group, p=0.49). In addition, no significant differences were observed for any of the other variables examined, including the WBC count, serum sodium level, CRP level, ALT and serum albumin level between the 2 groups. Analyses with an unpaired t test showed that the P value of NT-proBNP, Presepsin and PTX3 for IVIG resistance vs. responsiveness was 0.19, 0.04 and 0.001, respectively. PTX3 and Presepsin were significant. PTX3 exhibited a higher degree of significance than either NT-proBNP or Presepsin. The PTX3 values in the IVIG non-responders (n = 22) were significantly higher when compared to the IVIG responders (n= 46) (37.29±31.3 vs. 18.2±12.5; p=0.001). The threshold of >33.2 ng/ml PTX3 provided a sensitivity of 64.3% (95% CI: 53.35 to 81.83%), a specificity of 83.3% (95% CI: 54.35 to 95.95%), and a likelihood ratio as high as 12.2 for the diagnosis of “IVIG non-responder”.

**Table 3:** Demographic, laboratory characteristics of patients at the time of diagnosis in the IVIG responsive group, and the IVIG resistant group.

	IVIG res(+)	IVIG res(-)	P value
	(n = 46)	(n = 22)	
Age (m)	29.05±23.09	21.19±21.93	0.17
	[3-108]	[2-96]	
Male sex (%)*	0.50	0.67	0.18
Day of illness at the time of IVIG administration (d)	4.6±1.5	4.8±1.0	0.33
	[3-12]	[3-7]	
Sampling day of illness (d)	4.6±1.5	4.0±1.3	0.09
	[2-12]	[1-6]	
Complicated with CAL (%)*	0.50	0.41	0.49
WBC count (103/mL)	13790±3674	13405±4623	0.70
	[5500-24900]	[6000-23900]	
Sodium (mEq/L)	136.3±2.97	135.3±2.67	0.29
	[130-144]	[129-139]	
CRP (mg/dL)	6.71±4.15	7.32±3.24	0.55
	[1.26-20.55]	[1.7-15.64]	
D-Dimer (μg/mL)	2.01±0.85	2.53±1.49	0.22
	[1-4.33]	[0.82-5.13]	
ALT	75.8±15.1	116.2±39.0	0.24
	[7-422]	[8-807]	
ALB	3.81±0.33	3.80±0.30	0.87
	[2.9-4.5]	[3.1-4.3]	
NT-proBNP (pg/mL)	520.8±417.3	785.6±360.1	0.19
	[7.4-3437]	[6.2-2010]	
Presepsin (pg/mL)	489±217.6	943.8±463.3	0.04
	[103-2552]	[131-3437]	
PTX3 (ng/mL)	18.2±12.5	37.29±31.3	0.001
	[3.22-65.3]	[5.56-101.0]	

Presented as the mean ±SD with the 5th and 95th percentiles in square brackets. Mann-Whitney U test. *2-sided χ2 test. CAL means echo CAL, high echo density of the perivascular area of the coronary artery or coronary artery dilatation.

### Correlation of the PTX3, NT-proBNP and Presepsin Levels With IVIG Resistance

**Figure 2:**
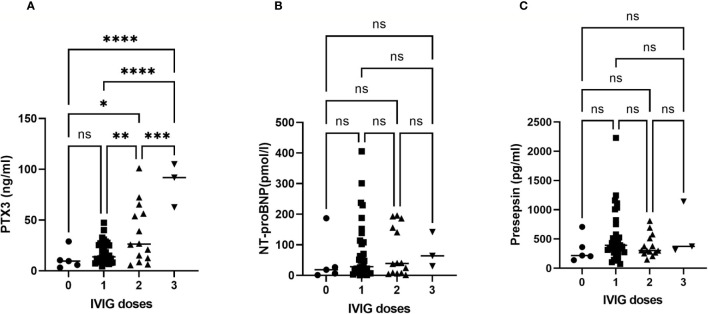
Correlation curve showing the dose of IVIG needed to resolve KD versus PTX3, NT-proBNP and Presepsin levels. The plasma PTX3 **(A)**, NT-proBNP **(B)** and Presepsin **(C)** levels are plotted against the dose of IVIG needed to resolve the KD. Zero indicates cases treated with aspirin only (non-IVIG). The PTX3 levels exhibited a significant correlation with IVIG doses required to resolve KD (y = 22.177 x - 4.2684, R² = 0.6402). ns, not significant; *p < 0.05, **p < 0.01, ***p < 0.005, ****p < 0.001.

**Figure 2** shows the plasma PTX3 value before IVIG treatment in patients who required 0, 1, 2, or 3 doses of IVIG to resolve the KD. The correlation of the IVIG dose number was obtained using the IVIG correlation coefficient which is a test of such a correlation. No significant correlation was found between the NT-proBNP or Presepsin level and the number of doses of IVIG needed to resolve inflammation in KD. The PTX3 levels in KD patients who required zero, one, two, or three doses of IVIG to resolve the KD were 12.5 ± 3.5, 21.0 ± 7.8, 35.6 ± 27.5, and 72.2 ± 32.8 ng/ml, respectively (significantly different according to one-way ANOVA, p < 0.05). The PTX3 level was also significantly correlated with the number of IVIG treatments needed to resolve fever. (p< 0.001). There was a strong correlation between the PTX3 value and the number of doses administered IVIG; y = 22.18 x - 4.27, R² =0.64.

### PTX3 and Coronary Abnormalities 

**Figure 3:**
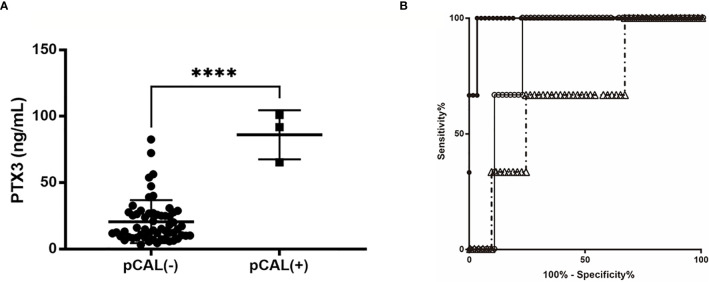
AUC and predictive power of the biomarker candidate levels at the time of admission. **(A)** Unpaired Student t-test. Comparison of the PTX3 level in patients with or without pCAL. ****p < 0.001. **(B)** ROC curves comparing the sensitivity and specificity of certain variables and CAL formation; the AUC values of PTX3, NT-proBNP and Presepsin were respectively 0.9885±0.014 (95% confidence interval [CI]: 0.961 to 1.00, p = 0.005), 0.8563±0.054 (95% CI: 0.7513 to 0.9614, p = 0.039), and 0.6667± 0.148 (95% CI: 0.3765 to 0.9568, p = 0.33). The cut-off value of 69 ng/ml to predict subsequent CAL had a specificity of 1.00, sensitivity of 0.98, and a likelihood ratio of 49.0 PTX3, filled circles •; NT-proBNP, open circles ○; Presepsin, open triangles △.

The patients with pCAL had higher PTX3 levels than those without pCAL (85 ± 8.4 vs. 22 ± 2.2 ng/ml, p < 0.0001). The NT-proBNP and Presepsin levels in KD patients with pCAL were not significantly higher than those without pCAL. All three cases with pCAL formation were IVIG non-responders (**Figure 3A**). The plasma PTX3 values on admission of these patients were 72.4, 91.8, and 101 ng/ml, the highest values in this study. **Figure 3B** shows the AUC and predictive power for pCAL of the candidate biomarker levels at the time of admission. The respective AUC value of PTX3, NT-proBNP and Presepsin was 0.99±0.014 (95% CI: 0.96 to 1.00, p=0.005), 0.86±0.054 (95% CI: 0.75 to 0.96, p=0.039), and 0.67± 0.15 (95% CI: 0.37 to 0.96, p=0.33). The cut-off value of 60.8 ng/ml that was used to predict subsequent pCAL had a sensitivity of 1.00 (95% CI: 29.24 to 100.0%), a specificity of 0.98 (95% CI: 88.09 to 99.58%), and a likelihood ratio of 29.0.

## Discussion

Prediction of CAL risk has become an important issue in KD because of recent advances in the therapeutic strategies available, such as prednisolone therapy (15) or treatment with a tumor necrosis factor-alpha antagonist (23), advances that enable stratification of the initial treatment for the patients with a high level of predicted risk. Thus, we carried out this collateral analysis to ascertain the diagnostic accuracy of PTX3, NT-proBNP and Presepsin in detecting CAL before the initial IVIG treatment.

NT-proBNP has been suggested to be a potential diagnostic biomarker for KD (24). NT-proBNP as a risk predicter of CAL after first being reported by Kaneko et al. (25), was suggested to be associated with the development of CAL. It was shown by a meta-analysis performed to ascertain the diagnostic value of NT-proBNP in detecting CAL in KD that the overall diagnostic sensitivity and specificity were 0.84 (95% CI: 0.78-0.89) and 0.71 (95% CI: 0.68-0.75), respectively. The area under the summary receiver operating characteristic curves (SROC) value was 0.8582 ± 0.0531 (24). The AUC value in our study was 0.6316 ± 0.08 (95% CI: 0.47 to 0.79), which is comparable to that of SROC in terms of meta-analysis. Both AUC values are still inferior to that of our PTX3 data of 0.9885±0.014 (95% CI: 0.96 to 1.00, p=0.005).

Presepsin is also known as a soluble cluster of differentiation (CD)14 subtype, which is a humoral risk stratification marker for systemic inflammatory response syndrome and sepsis. The results of many clinical studies have indicated that Presepsin is a useful biomarker not only for early diagnosis, but also for risk stratification and prognosis prediction for sepsis in adults (26) as well as children (27). The present study shows that Presepsin was, as the case in sepsis (28), inferior to PTX3 in predicting IVIG resistance and**/**or CAL formation.

The pathophysiology of the acute phase of Kawasaki disease is characterized by excessive activation of the innate immune system and is accompanied by an increase in inflammatory cytokines and chemokines (29). Furthermore, it is thought that these factors trigger the induction of the fever, acute phase protein production in the blood, neutrophil increase, and vasculitis. PTX3 is a prototypic soluble pattern recognition receptor in the innate immune system that is expressed at sites of inflammation and is involved in regulation of the tissue homeostasis (18). PTX3 has also been studied as a diagnostic biomarker for sepsis. PTX3 levels are low under normal conditions (<2 ng/ml in adults, 1.24 ng/ml [0.87–2.08] in children) (30), but the plasma PTX3 concentrations increase in certain conditions such as sepsis, small-vessel vasculitis (31), and acute myocardial infarction (32). In sepsis, PTX3 may serve as an early marker of the severity and outcome of the disease. PTX3 is correlated with the severity of sepsis in febrile patients presenting with the disease in hospital emergency rooms (33). PTX3 levels also elevated in patients with systemic inflammation (median = 71.3 ng/ml) (34). A role for PTX3 as a sensitive marker of vascular inflammation has also been proposed (18, 35). The innate immune system, which is considered to be involved in the pathogenesis of KD, includes a humoral arm such as PTX3 that is complementary to cellular recognition and effector function (18).

In this study, we observed the following in patients with KD. a) The plasma PTX3 and NT-proBNP values were significantly increased compared with these values in non KD febrile patients determined before IVIG. Using 25.7 ng/ml as the optimal cut-off, we identified PTX3 as an excellent biomarker for the purpose of differentiating KD cases from non-KD cases, including subjects highly suspected of KD. b) Patients with acute early coronary lesions presented with a greater increase in the PTX3 plasma values than patients without any acute early coronary artery lesions. c) Patients who had PTX3 plasma values exceeding 33.2 ng/ml exhibited increased risk of IVIG resistance. d) A significant correlation was found between the levels of PTX3 and the corresponding number of doses of IVIG treatment needed to resolve the inflammation caused by KD (p < 0.01). e) The cut-off value of 60.8 ng/ml to predict subsequent CAL had a specificity of 1.00, sensitivity of 0.98, and a likelihood ratio of 29.0.

Katsube et al. reported the PTX3 level in 56 patients with KD. They determined that the PTX3 levels in unresponsive cases (n=7) were significantly higher than in responsive cases (n=49) (55.3 vs. 18.9; p<0.01). There were two cases of CAL among the unresponsive cases, and the PTX3 values in these cases were 63.7 and 89.3 ng/ml (36). These PTX3 values are similar to those of the three CAL cases in our study. The sensitivity and specificity of PTX3 in predicting IVIG unresponsiveness were 85.7 and 95.9 % when the PTX3 cut-off value was set at 40 ng/ml (36). In our study, the cut-off value of 60.8 ng/ml used to predict subsequent CAL had a specificity of 1.00, sensitivity of 0.98, and a likelihood ratio of 29.0. A very similar predictive power was observed in an independent report (37). We also observe that the plasma PTX3 level was elevated in KD before IVIG to 33.4 ± 7.3, more than 10 times higher than the normal value in controls (2.7 ± 1.0) (30), and returned to an improved but still abnormal level of 9.64 ± 3.61 during convalescence. Katsube et al also reported the sustained PTX3 elevation that implies continuing silent vasculitis even in one month after the onset of KD.

Recently, Ching et al. reported that significantly higher serum PTX3 levels in patients with CAL compared to patients without CAL, as well as IVIG resistance during the acute phase of KD. They concluded PTX3 as a sensitive marker for coronary artery dilation in KD rather than IVIG resistance (38). Our observation is consistent with them regarding to coronary artery dilatation and IVIG resistance. In this study, we found that the elevated PTX3 level correlated with the number of doses administered IVIG. In our cohort, there were three cases of pCAL formation with highly elevated plasma PTX3 level. These results suggested that the PTX3 is a good marker for pCAL formation and the severity of KD. Our results further strengthen their recommendation to utilize PTX3 in assessing KD patients.

The limitations of this study include limited sample size, particularly within the pCAL group. However, the results clearly demonstrate a significant elevation of the PTX3 level in KD patients that is correlated with required IVIG doses for responsiveness. These data further suggest a specific role for PTX3 in KD disease severity as well as the treatment response. Future studies should focus on investigating the underlying mechanisms by which PTX3 and other innate immunity proteins are involved in KD pathogenesis and CAL development.

## Conclusions

It is demonstrated that PTX3 is highly useful in the diagnosis of KD. The PTX3 level before IVIG was a strong and sensitive predictor of IVIG non-responsiveness and subsequent CAL formation, and may help identify high-risk patients requiring additional second-line therapy other than repeated IVIG treatment alone. According to these results, the prognostic value of PTX3 as a single biomarker is the best available when compared with the currently used risk scoring systems (e.g. the Kobayashi Score) (11), or other proposed predictors such as NT-proBNP or Presepsin. A particularly high level of PTX3 is also an independent predictor of CAL formation after fever resolution.

## Data Availability Statement

The raw data supporting the conclusions of this article will be made available from the corresponding author TK on request.

## Ethics Statement

The studies involving human participants were reviewed and approved by Institutional Review Board of the Aichi Medical University Hospital. Written informed consent to participate in this study was obtained from the participants’ legal guardian/next of kin.

## Author Contributions

TK participated in the design of the study, collected data, interpreted the data, did the statistical analysis, and wrote the initial draft report. TO performed the NT-pro BNP assay, collected data, interpreted data, and revised the report. AO supervised his assay. RB performed and read echocardiography and defined the final outcome of the KD patient for any coronary artery lesions. YK performed the PTX3 and Presepsin assay. YY and HM supervised his assay. KD and TH participated in the design of the study and reviewed the manuscript, and approved the final manuscript as submitted. All authors contributed to the article and approved the submitted version.

## Funding

This work was supported by Tokai Foundation for Technology (to TK) Toyohashi, Aichi, Japan, and Research grants project number AS242Z02588P (to TK and TH) from the New Energy and Industrial Technology Development Organization, Minister of Economy, Trade, and Industry, Japan. This work was supported by JSPS KAKENHI grant number JP16K08422 (to TK, TM, HM, KD, and TH).

## Conflict of Interest

The authors declare that the research was conducted in the absence of any commercial or financial relationships that could be construed as a potential conflict of interest.

## Abbreviations

AUC, area under curve; CAA, coronary artery aneurysm; CAL, coronary artery lesion; IVIG, intravenous gamma-globulin; KD, Kawasaki disease; ROC curve, receiver operating characteristic curve.
